# D-Dimer as a Prognostic Indicator in Critically Ill Patients Hospitalized With COVID-19 in Leishenshan Hospital, Wuhan, China

**DOI:** 10.3389/fphar.2020.600592

**Published:** 2020-12-21

**Authors:** Jinpeng Li, Zeming Liu, Gaosong Wu, Meilin Yi, Yongfeng Chen, Kun Li, Xiaoming Xu, Linqi Xiao, Qian Wu, Jincao Chen, Xiaohui Wu

**Affiliations:** ^1^ Department of Thyroid and Breast Surgery, Zhongnan Hospital of Wuhan University, Wuhan, China; ^2^ Department of Plastic Surgery, Zhongnan Hospital of Wuhan University, Wuhan, China; ^3^ Department of Burn and Plastic Surgery, College of Traditional Chinese Medicine, Three Gorges University and Yichang Hospital of Traditional Chinese Medicine, Yichang, China; ^4^ Medical Department, Zhongnan Hospital of Wuhan University, Wuhan, China; ^5^ Department of Hepatobiliary and Pancreatic Surgery, Zhongnan Hospital of Wuhan University, Wuhan, China; ^6^ Department of Medical Records and Statistics, The Central Hospital of Wuhan, Tongji Medical College, Huazhong University of Science and Technology, Wuhan, China; ^7^ Hospital Management Institute, Zhongnan Hospital of Wuhan University, Wuhan, China; ^8^ Department of Neurosurgery, Zhongnan Hospital of Wuhan University, Wuhan, China

**Keywords:** SARS–CoV–2, prognostic indicator, critically ill patients, COVID-19, d-dimer

## Abstract

**Background:** D-dimer is a small protein fragment and high levels of D-dimer have been associated with increased mortality in patients presenting to emergency departments with infection. Previous studies have reported increased levels of D-dimer in COVID-19; however, it is unclear whether an increased D-dimer level provides early warning of poor prognosis. Therefore, this study aimed to assess the usefulness of D-dimer as an early indicator of prognosis in patients with coronavirus disease (COVID-19).

**Methods:** We conducted a retrospective study of patients with COVID-19 admitted to Leishenshan Hospital in Wuhan, China, from February 15 to March 30, 2020. The final date of follow-up was April 11, 2020.

**Results:** Of the 1,643 patients with COVID-19, 691 had elevated D-dimer levels. Their median age was 65 years. Of the patients with elevated D-dimer levels, 45% had comorbidities, with cardiovascular disease (205 [29.7%]) being the most common. Patients with elevated D-dimer were more likely to require treatment with high-flow oxygen, anticoagulation, antibiotics, and admission to the intensive care unit They were also more likely to have increased interleukin-6, monocytes, and lymphocytes. Patients with elevated D-dimer levels had significantly higher mortality than those with normal or low D-dimer levels.

**Conclusion:** In patients with COVID-19, elevated D-dimer was associated with abnormal immunity, underlying disease, increased disease severity, and increased mortality. Taken together, D-dimer may be a marker for the early warning of disease severity and increased risk of death. These findings provide insights into the potential risk of elevated D-dimer in patients with COVID-19.

## Introduction

A cluster of acute respiratory illness subsequently recognized as coronavirus disease (COVID-19), occurred in Wuhan, China, in December 2019, (Epidemiology Working Group for NCIP Epidemic Response, Chinese Center for Disease Control and Prevention, 2020; Hui et al., 2020) and it subsequently spread to all regions of the world. Between December 2019 and April 27, 2020, almost three million people were diagnosed with COVID-19 worldwide. In addition to Wuhan in China, Korea, Italy, the United States, France, Germany, Iran, and the United Kingdom have experienced major national epidemics (Huang et al., 2020). Common symptoms of COVID-19 include fever, dyspnea, dry cough, fatigue, and myalgia. Common laboratory findings include normal or decreased leukocyte counts, lymphopenia, and increased C-reactive protein, and radiography usually shows evidence of pneumonia (Huang et al., 2020). Wand et al. reported that among 138 hospitalized patients with COVID-19 pneumonia, 26% require intensive care unit (ICU) care, and the mortality is 4.3% (Wang et al., 2020).

In the patients with COVID-19 pneumonia, the mortality rate increases with age. Older people are more likely to have comorbidities such as diabetes mellitus, hypertension, cardiac disease, and cancer. A recent study reported that older age was associated with greater risk of development of acute respiratory distress syndrome and death, which was probably due to lower levels of immunity in general (Ciceri et al., 2020; Wu et al., 2020). Additionally, hypertension was reported to be the most common comorbidity in an analysis of fatal cases of COVID-19 pneumonia, followed by diabetes and coronary heart disease (Varga et al., 2020; Zhou et al., 2020). A higher D-dimer level (>1 μg/ml) appeared to be associated with an increased risk of death.

D-dimer is a fibrin degradation product, a small protein fragment that is released into the blood when a blood clot is degraded by fibrinolysis (Adam et al., 2009). A diagnosis of thrombosis can be made by determining the D-dimer concentration: the D-dimer level is increased when the coagulation system is activated by thrombosis or disseminated intravascular coagulation. A high level of D-dimer may also be a sign of deep venous thrombosis, pulmonary embolism, or disseminated intravascular coagulation (DIC) (Ranasinghe and Bonser, 2010). Zhou et al. reported that a high level of D-dimer may help in the early clinical diagnosis of COVID-19 (Zhou et al., 2020). Furthermore, in the 2003 severe acute respiratory syndrome (SARS) epidemic, most patients had thrombocytopenia and elevated levels of D-dimer (Tan et al., 2005). Similarly, elevated D-dimer levels have also been reported in patients with Ebola (Smither et al., 2019). Although there have been some reports on increased levels of D-dimer in COVID-19 patients, it is unclear whether an increased D-dimer level is an early warning of a poor prognosis.

In this study we analyzed the D-dimer levels in COVID-19 patients admitted to Leishenshan Hospital in Wuhan, China, up to March 30, 2020. The aim of the analysis was to determine the association between elevated D-dimer levels and clinical signs, other laboratory findings, and the associated treatment in patients hospitalized with COVID-19.

## Methods

### Study Design and Participants

This retrospective review of medical records included 1643 COVID-19 patients admitted to Leishenshan Hospital in Wuhan, China, from February 15 to March 30, 2020. The final date of follow-up was April 11, 2020. All patients were diagnosed with COVID-19 according to the World Health Organization interim guidance and the COVID-19 Prevention and Control guidelines (seventh edition) published by the National Health Commission of China. The patients provided oral consent. This study was approved by the Research Ethics Committee of Wuhan University Zhongnan Hospital (approval number 2020004).

### Data Collection

We reviewed the clinical and laboratory findings, demographic data, treatment, and chest computed tomography (CT) scans of all 1643 COVID patients admitted to Leishenshan Hospital during the study period. All data were collected from electronic medical records using a customized data collection form. Two physicians at Leishenshan Hospital collected and analyzed all data. All the laboratory outcomes were measured in the first time on admission. The methods for laboratory diagnosis of COVID-19 were the same as those reported previously (Huang et al., 2020). In brief, throat swab samples were collected from patients with suspected COVID-19 pneumonia and immediately tested for severe acute respiratory syndrome coronavirus 2 (SARS-CoV-2) RNA. All patients had at least three throat swab samples collected. The RT-PCR assay for the detection of SARS-CoV-2 nucleic acid was performed according to the manufacturer’s protocol (BioGerm, Shanghai Berger Medical Technology Company, Pudong New District, China). Venous blood samples were collected from the antecubital fossa and tested for immunoglobulin G (IgG) and immunoglobulin M (IgM) antibodies to SARS-CoV-2.

### Statistical Analysis

Continuous variables with a normal distribution were reported as the mean ± standard deviation; otherwise, they were reported as the median and interquartile range (IQR). Categorical variables were described as frequencies and percentages. Two-sample t-tests or Mann-Whitney tests were used to test whether there were any significant differences in the mean of continuous variables between the higher and lower D-dimer groups. The proportions of categorical variables in these two groups were compared using the chi-square test or Fisher’s exact test when the cell size was small. Univariate and multivariate logistic regression analyses were performed to determine the relationship between D-Dimer level and the prognosis of COVID-19 patients. Patient survival curves and the cumulative hazard function for COVID-19 progression in both groups were analyzed using Kaplan-Meier analyses with log-rank tests. To further detect the relationship between the CT score in different D-Dimer level and survival days, curve fitting analysis was performed. All the statistical analyses were performed using SPSS Version 23.0 (IBM Corp, Armonk, NY, United States). Two-sided *p*-values < 0.05 were considered to indicate statistical significance.

## Results

A total of 1,643 patients with COVID-19 were admitted to Leishenshan Hospital from February 15 to March 30, 2020. The median age of the patients was 59.0 years old (IQR: 49.0–68.0 years), and 782 (47.6%) were male. The common clinical symptoms at onset of illness included fever and/or fatigue, and respiratory system, digestive system and nervous system signs and symptoms similar to cases of COVID-19 reported previously. Of the patients, 490 (29.8%) had comorbidities including cardiovascular disease (19.5%), endocrine disease (diabetes) (7.7%), pulmonary disease (5.2%), neurological disease (3.1%), malignancy (3.7%), and digestive system disease (2.5%). Elevated lymphocyte and decreased red blood cell count, hemoglobin, and albumin were observed in the patients. Among 1,643 patients, 212 of 581 patients showed a positive result for SARS-CoV-19 IgM, while 505 of 552 patients displayed a positive result for SARS-CoV-19 IgG (Table 1). Abnormal coagulation system did not occur in the 1,643 patients.

**TABLE 1:** Demographic characteristics and symptoms of 1,643 patients with COVID-19.

Covariates	Levels	All patients (*n* = 1,643), *n* (%)	Normal D-Dimer (*n* = 952), *n* (%)	Elevated D-Dimer (*n* = 691), *n* (%)	*p* Value
Gender					0.491
	Female	861 (52.4)	492 (51.7)	369 (53.4)	
	Male	782 (47.6)	460 (48.3)	322 (46.6)	
Age, median (IQR)		59 (49–68)	55 (44–64)	65 (56–72)	<0.001
Any comorbidity		490 (29.8)	179 (18.8)	311 (45.0)	<0.001
	Cardiovascular diseases	321 (19.5)	116 (12.2)	205 (29.7)	<0.001
	Pulmonary diseases	85 (5.2)	37 (3.9)	48 (6.9)	0.006
	Endocrine diseases	126 (7.7)	43 (4.5)	83 (12)	<0.001
	Malignancy	60 (3.7)	22 (2.3)	38 (5.5)	0.001
	Digest systemdiseases	41 (2.5)	15 (1.6)	26 (3.8)	0.005
	Neurological diseases	51 (3.1)	9 (0.9)	42 (6.1)	0.005
Initial symptoms, n (%)					
	Fever or fatigue	625 (38)	262 (27.5)	363 (52.5)	<0.001
	Respiratory symptoms	639 (38.9)	270 (28.4)	369 (53.4)	<0.001
	Digestive symptoms	85 (5.2)	40 (4.2)	45 (6.5)	0.037
	Neurological symptoms	26 (1.6)	9 (0.9)	17 (2.5)	0.015
	Other	25 (1.5)	11 (1.2)	14 (2.0)	0.155

Of the 1,643 patients, 691 had elevated D-dimer levels (>0.5 mg/L). The median age was 65 years old (IQR: 56.0–72.0 years), 322 (46.6%) were male (Table 1). Of 691 patients who displayed a high level of D-dimer, the most common clinical symptoms at the onset of illness included fever or dry cough (363 [52.5%]), respiratory system (369 [53.4%]), digestive system (45 [6.5%]), and nervous system (17 [2.5%]) (Table 1). Comorbidities were present in 45% patients with high D-dimer levels, with cardiovascular disease (205 [29.7%]) be the most common comorbidity, followed by diabetes (83 [12%]), hypertension (48 [6.9%]), neurological diseases (42 [6.1%]) and cancers (38 [5.5%]) (Table 1). Furthermore, 83 (12%) patients had lymphocytopenia and 222 (32.1%) patients had elevated lymphocyte counts. The decreased erythrocyte (547 [79.2%]), hemoglobin (523 [75.7%]) and albumin (569 [86.3%]) presented in the patients. About half of patients had decreased creatinine (Table 2). We next analyzed the coagulation system, and we found that a high level of fibrinogen and decreased activated partial thromboplastin time and thrombin time accompanied by high level of D-dimer (Table 3). These findings suggested that the abnormal coagulation system and decreased kidney function occurred with high levels of D-dimer.

**TABLE 2:** Laboratory test results of 1,643 patients with COVID-19.

Covariate	All patients (*n* = 1,643) median (IQR)/*n* (%)	Normal D-Dimer (*n* = 952) median (IQR)/*n* (%)	Elevated D-Dimer (*n* = 691) median (IQR)/*n* (%)	*p* Value	References range
Leukocyte count, × 10⁹/L	5.7 (4.7–6.9)	5.7 (4.8–6.8)	5.8 (4.7–7.1)	0.533	3.5–9.5
3.5–9.5	1,458 (88.8)	874 (91.9)	584 (84.5)	<0.001	
<3.5	100 (6.1)	48 (5.0)	52 (7.5)		
>9.5	84 (5.1)	29 (3.0)	55 (8.0)		
Neutrophil count, × 10⁹/L	3.3 (2.5–4.3)	3.2 (2.5–4.0)	3.5 (2.6–4.7)	<0.001	1.8–6.3
1.8–6.3	1,429 (87.0)	870 (91.5)	559 (80.9)	<0.001	
<1.8	106 (6.5)	57 (6.0)	49 (7.1)		
>6.3	107 (6.5)	24 (2.5)	83 (12.0)		
Lymphocyte count, × 10⁹/L	1.6 (1.2–2.0)	1.7 (1.4–2.1)	1.4 (1.0–1.8)	<0.001	1.1–3.2
1.1–3.2	955 (58.2)	569 (59.8)	386 (55.9)	<0.001	
<1.1	122 (7.4)	39 (4.1)	83 (12.0)		
>3.2	565 (34.4)	343 (36.1)	222 (32.1)		
Erythrocyte count, × 10^12^/L	4.1 (3.8–4.5)	4.3 (4.0–4.6)	3.9 (3.5–4.2)	<0.001	4.3–5.8
4.3–5.8	573 (34.9)	431 (45.3)	142 (20.5)	<0.001	
<4.3	1,060 (64.6)	513 (53.9)	547 (79.2)		
>5.8	9 (0.5)	7 (0.7)	2 (0.3)		
Monocyte count, × 10⁹/L	0.5 (0.4–0.6)	0.5 (0.4–0.6)	0.5 (0.4–0.7)	0.016	0.1–0.6
0.1–0.6	335 (20.4)	165 (17.4)	170 (24.6)	0.001	
<0.1	2 (0.10)	1 (0.1)	1 (0.1)		
>0.6	1,305 (79.50)	785 (82.5)	520 (75.3)		
Hemoglobin, g/L	125.5 (115.0–137.0)	130.0 (121.0–140.0)	118.0 (107.0–129.0)	<0.001	130.0–175.0
130.0–175.0	641 (39.0)	475 (49.9)	166 (24.0)	<0.001	
<130.0	997 (60.7)	474 (49.9)	523 (75.7)		
>175.0	4 (0.2)	2 (0.2)	2 (0.3)		
Platelet count, × 10⁹/L	229.0 (186.3–279.0)	227.0 (188.0–274.0)	232.0 (185.0–294.0)	0.200	125.0–350.0
125.0–350.0	1,425 (86.8)	856 (90.0)	569 (82.3)	<0.001	
<125.0	68 (4.1)	26 (2.7)	42 (6.1)		
>350.0	149 (9.1)	69 (7.3)	80 (11.6)		
Albumin, g/L	37.7 (35.0–40.1)	38.8 (36.9–40.8)	35.4 (32.7–38.4)	<0.001	40.0–55.0
40–55	411 (25.8)	321 (34.4)	90 (13.7)	<0.001	
<40	1,182 (74.2)	613 (65.6)	569 (86.3)		
Alanine aminotransferase, U/L	23 (15–37)	23 (15–39)	22 (14–35)	0.038	9–50
9–50	1,269 (79.7)	748 (80.1)	521 (79.1)	0.024	
<9	90 (5.6)	41 (4.4)	49 (7.4)		
>50	234 (14.7)	145 (15.5)	89 (13.5)		
Aspartate aminotransferase, U/L	20 (16–27)	20 (16–26)	20 (16–28)	0.495	15–40
15–40	1,159 (72.8)	696 (74.5)	463 (70.3)	0.021	
<15	294 (18.5)	171 (18.3)	123 (18.7)		
>40	140 (8.8)	67 (7.2)	73 (11.1)		
Total bilirubin, μmol/L	9.2 (7.0–12.2)	9.2 (7.1–12.1)	9.3 (6.8–12.2)	0.675	5.0–21.0
5.0–21.0	1,419 (89.1)	855 (91.5)	564 (85.6)	0.001	
<5.0	109 (6.8)	50 (5.4)	59 (9.0)		
>21.0	65 (4.1)	29 (3.1)	36 (5.5)		
Creatinine, μmol/L	64.3 (54.5–75.8)	63.8 (54.2–74.4)	65.1 (54.7–78.9)	0.013	64.0–104.0
64.0–104.0	715 (44.8)	439 (46.9)	276 (41.8)	<0.001	
<64.0	785 (49.2)	476 (50.9)	309 (46.8)		
>104.0	96 (6.0)	21 (2.2)	75 (11.4)		
Procalcitonin, ng/mL	0.04 (0.03–0.06)	0.03 (0.02–0.04)	0.04 (0.03–0.08)	<0.001	<0.05
<0.05	902 (64.8)	600 (75.4)	302 (50.6)		
≥0.05	491 (35.2)	196 (24.6)	295 (49.4)		
Interleukin-6, pg/mL	1.5 (1.5–4.2)	1.5 (1.5–2.3)	3.0 (1.5–8.0)	<0.001	0–7.0
0–7.0	571 (83.0)	348 (92.6)	223 (71.5)	<0.001	
>7.0	117 (17.0)	28 (7.4)	89 (28.5)		
SARS-CoV-19 IgM				0.302	-
NO	369 (63.5)	189 (61.6)	180 (65.7)		
YES	212 (36.5)	118 (38.4)	94 (34.3)		
SARS-CoV-19 IgG				0.426	-
NO	47 (8.5)	22 (7.6)	25 (9.5)		
YES	505 (91.5)	267 (92.4)	238 (90.5)		

**TABLE 3:** Blood coagulation test results of 1,643 patients with COVID-19.

Covariate	All patients (*n* = 1,643) median (IQR)/*n* (%)	Normal D-Dimer (*n* = 952) median (IQR)/*n* (%)	Elevated D-Dimer (*n* = 691) median (IQR)/*n* (%)	*p* Value	References range
Prothrombin time, s	11.3 (10.9–11.8)	11.2 (10.8–11.6)	11.4 (11.0–12.1)	<0.001	9.4–12.5
9.4–12.5	1,511 (92.0)	913 (95.9)	598 (86.5)	<0.001	
<9.4	1 (0.1)	1 (0.1)	0 (0)		
>12.5	131 (8.0)	38 (4.0)	93 (13.5)		
International normalized ratio	1.0 (0.9–1.0)	1.0 (0.9–1.0)	1.0 (0.9–1.1)	<0.001	0.8–1.3
0.8–1.3	1,616 (98.4)	945 (99.3)	671 (97.1)	<0.001	
<0.8	1 (0.1)	1 (0.1)	0 (0)		
>1.3	26 (1.6)	6 (0.6)	20 (2.9)		
Activated partial thromboplastin time, s	27.2 (24.6–30.4)	27.2 (24.7–30.3)	27.2 (24.3–30.9)	0.673	25.1–36.5
25.1–36.5	1,070 (65.1)	638 (67.0)	432 (62.5)	0.147	
<25.1	485 (29.5)	268 (28.2)	217 (31.4)		
>36.5	88 (5.4)	46 (4.8)	42 (6.1)		
Fibrinogen, (g/L)	2.95 (2.51–3.73)	2.80 (2.40–3.30)	3.43 (2.71–4.08)	<0.001	2.38–4.98
2.38–4.98	1,038 (63.2)	639 (67.1)	399 (57.7)	<0.001	
<2.38	316 (19.2)	227 (23.8)	89 (12.9)		
>4.98	289 (17.6)	86 (9)	203 (29.4)		
Thrombin time, s	17.6 (17.0–18.4)	17.7 (17.2–18.5)	17.4 (16.7–18.3)	<0.001	10.3–16.6
10.3–16.6	248 (15.1)	88 (9.2)	160 (23.2)	<0.001	
<10.3	1,341 (81.6)	831 (87.3)	510 (73.8)		
>16.6	54 (3.3)	33 (3.5)	21 (3.0)		
D-dimer, g/L	0.39 (0.21–0.91)	0.24 (0.16–0.33)	1.08 (0.74–2.2)	<0.001	0–0.50

**FIGURE 1:**
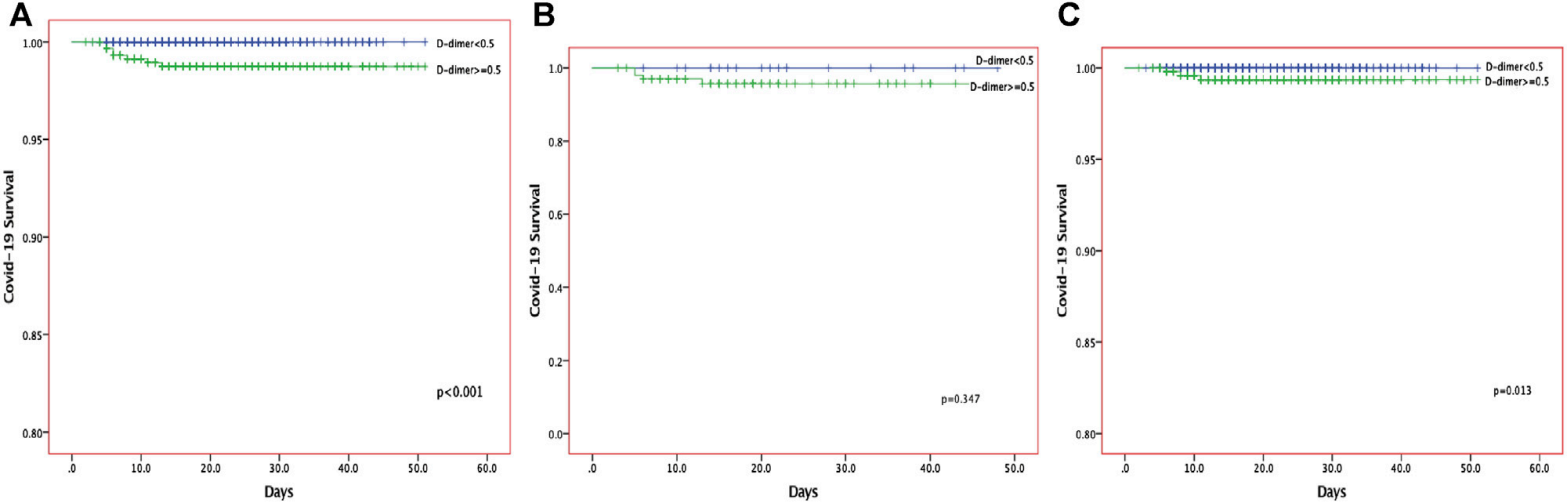
The Kaplan-Meier curves of the survival of COVID-19 patients for all the COVID-19 patients **(A)**, anticoagulant group **(B)** and non-anticoagulant group **(C)**.

When compared with patients with normal D-dimer levels, patients with elevated D-dimer levels were more likely to receive t antibiotic therapy (249 [36%]), anticoagulants (121 [17.5%]), and corticosteroids (72 [10.4%]). Additionally, more patients needed high-flow oxygen support (31 [21.2%]). Five patients required invasive mechanical ventilation, and one patient treated by extracorporeal membrane oxygenation (ECMO), all of whom died. A high level of D-dimer was associated with the severity of the disease on admission. There was a significant difference in survival between patients with normal D-dimer and those with elevated D-dimer (Figure 1). The number of survivors among those with elevated D-dimer decreased significantly with time. Similarly, in multivariate logistic regression analysis, an elevated D-dimer was associated with a significantly higher mortality rate (HR:0.88 95%CI:0.58–1.33, P0.53, Table 4). However, both univariate analysis (HR:3.77, 95% CI: 3.06–4.64, *p* < 0.001) and multivariate analysis (HR:3.04, 95%CI:1.674–5.507, *p* < 0.001) show elevated D-dimer was risk factor predict the disease severity (Table 5).

**TABLE 4:** The risk of elevated D-Dimer for the disease mortality of COVID-19.

Group		Logistic regression analysis			
		HR	95% CI		*p* Value
Univariate analysis	D-dimer<0.5 g/L	Ref			
	D-dimer ≥ 0.5 g/L	3.765	3.057	4.637	<0.001
Multivariate analysis^a^	D-dimer < 0.5 g/L	Ref			
	D-dimer ≥ 0.5 g/L	0.876	0.579	1.325	0.530

a Adjust for Age, History of cardiovascular disease, Leukocyte count, Platelet count, Lymphocyte count, Total bilirubin, Creatinine, Oxygen support.

**FIGURE 2:**
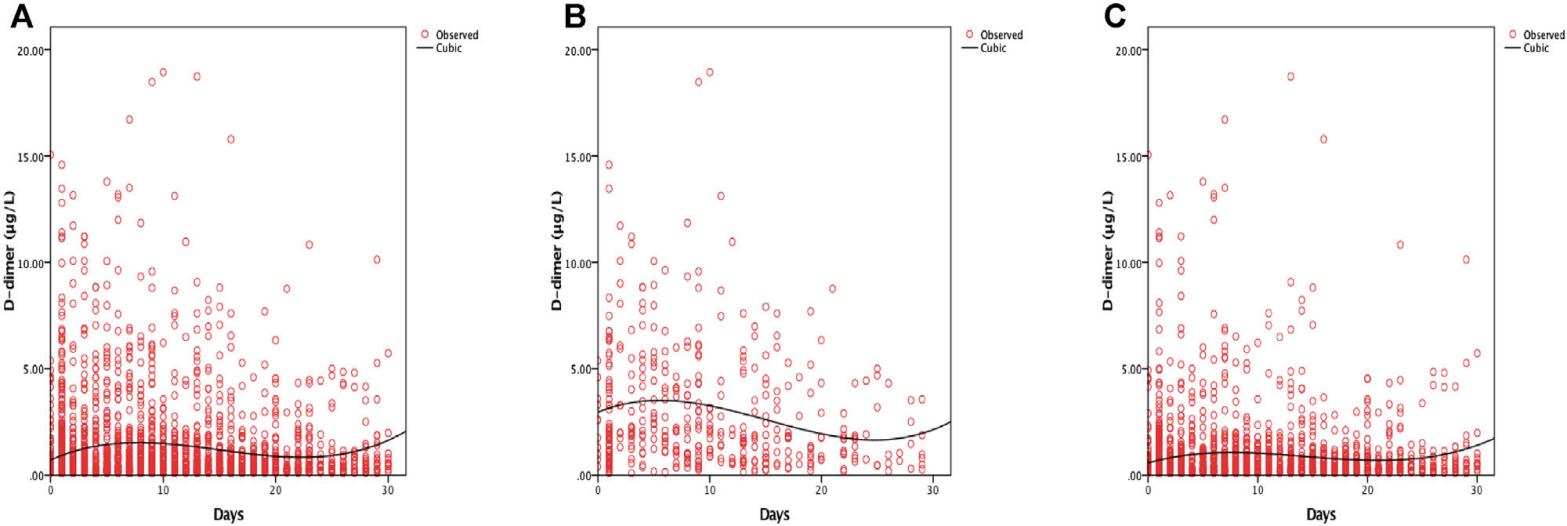
Curve fitting analysis for all the COVID-19 patients **(A)**, anticoagulant group **(B)** and non-anticoagulant group **(C)**.

Of the 1,643 patients, 134 (8.2%) received anticoagulant treatment; 121 of 134 patients had a high level of D-dimer (Table 6). Of the patients, 394 required oxygen support including low-flow nasal cannula, non-invasive ventilation or high-flow nasal cannula, invasive mechanical ventilation and ECMO; 136 of 394 showed increased D-dimer levels (Table 6). D-dimer levels peaked 10 days after admission. However, anticoagulation therapy significantly decreased that time (7 days) (Figure 2).

**TABLE 5:** The risk of elevated D-Dimer for the disease severity of COVID-19.

Group		Logistic regression analysis			
		HR	95% CI		*p* Value
Univariate analysis	D-dimer < 0.5 g/L	Ref			
	D-dimer ≥ 0.5 g/L	3.765	3.057	4.637	<0.001
Multivariate analysis^a^	D-dimer < 0.5 g/L	Ref			
	D-dimer ≥ 0.5 g/L	3.036	1.674	5.507	<0.001

a Adjust for Age, Leukocyte count, Platelet count, Lymphocyte count, Total bilirubin, Creatinine, Oxygen support.

**TABLE 6:** Clinical treatment and outcomes of 1,643 patients with COVID-19.

Covariates	Levels	All patients (*n* = 1,643) *n* (%)	Normal D-Dimer (*n* = 952) *n* (%)	Elevated D-Dimer (*n* = 691) *n* (%)	*p* Value
Drugs					
	Antibiotic	461 (28.1)	212 (22.3)	249 (36.0)	<0.001
	Antiviral drugs	770 (46.9)	431 (45.3)	339 (49.1)	0.129
	Antimalarial drugs	130 (7.9)	80 (8.4)	50 (7.2)	0.387
	Anticoagulants	134 (8.2)	13 (1.4)	121 (17.5)	<0.001
	Corticosteroid	96 (5.8)	24 (2.5)	72 (10.4)	<0.001
	Vitamin C	241 (14.7)	140 (14.7)	101 (14.6)	0.960
	Traditional Chinese medicine	1,406 (85.6)	812 (85.3)	594 (86)	0.704
Oxygen support					<0.001
	Low-flow nasal cannula	248 (84.4)	139 (93.9)	109 (74.7)	
	Non-invasive ventilation or high-flow nasal cannula	40 (13.6)	9 (6.1)	31 (21.2)	
	Invasive mechanical ventilation	5 (1.7)	0 (0)	5 (3.4)	
	ECMO	1 (0.3)	0 (0)	1 (0.7)	
CT scores	1–4	77 (41.2)	20 (51.3)	57 (38.5)	0.149
	5–7	110 (58.8)	19 (48.7)	91 (61.5)	
Disease progression					<0.001
	Stableness/Hospitalization	15 (0.9)	3 (0.3)	12 (1.8)	
	Improvement/Recover	1,594 (98.2)	948 (99.7)	646 (96.1)	
	Death	14 (0.9)	0 (0)	14 (2.1)	
Days in hospital, median (IQR)		18 (13–24)	17 (12–22)	20 (15–28)	<0.001
ICU Care		35 (2.1)	1 (0.1)	34 (4.9)	0.205
Severity on admission					
	Mild	771 (48.0)	529 (56.3)	242 (36.3)	<0.001
	Moderate	525 (32.7)	317 (33.8)	208 (31.2)	
	Severe	284 (17.7)	91 (9.7)	193 (29.0)	
	Critical	25 (1.6)	2 (0.2)	23 (3.5)	
Severity at worst					
	Mild	813 (49.7)	598 (63.0)	215 (31.3)	<0.001
	Moderate	1 (0.1)	1 (0.1)	0 (0)	
	Severe	774 (47.3)	346 (36.5)	428 (62.2)	
	Critical	49 (3.0)	4 (0.4)	45 (6.5)	

To keep track of the patient's progress, we dynamically observed the multiple patchy ground-glass shadows when performing repeated chest CT scans. The CT score was divided into mild-to-moderate (Score 1–4) and severe (Score 5–7) (Table 6). Of the 187 patients, 110 (58.8%) displayed serious lung inflammation; almost 83% of them showed a high level of D-dimer (Table 6). The 691 patients with high D-dimer levels spent a median of 20 days in hospital (IQR: 15–28 days), which twas higher than the median length of stay of patients with normal D-dime levels. Of the 216 (32.5%) patients with a severe condition on admission, the incidence rate of severe cases increased to 68.7% after increased hospitalization time (Table 6). Further, 64% of them had comorbidities, such as cardiovascular disease and endocrine disease. The median age was 65 years old (IQR 56–72), and they were older than common COVID-19 patients. Common clinical symptoms such as fever/fatigue and respiratory symptom were also investigated in patients who showed elevated D-dimer (Table 1). Furthermore, more patients who had elevated D-dimer had higher severity, which were required the high-flow oxygen support even invasive mechanical ventilation or ECMO (Table 6). These findings suggest the synchronous performance between radiography features and clinical symptom in elevated D-dimer patients.

## Discussion

In this retrospective cohort study, we analyzed the most common risk factor of patients with COVID-19 who were in ICU treatment in Leishenshan, Wuhan, China; elevated D-dimer was observed in 691 out of 1,643 patients. We then summarized the associated clinical course, basic disease, and final outcome. 1) Of 1,643 patients in Leishenshan Hospital, Wuhan, 691 showed elevated levels of D-dimer. Common clinical symptoms such as fever/fatigue and respiratory symptom were observed in patients who showed elevated D-dimer. Elevated lymphocyte and monocyte counts were observed in a higher number of patients. 2) Comorbidities were more common in patients who displayed high levels of D-dimer. 3) More critically ill patients and higher mortality were observed in elevated D-dimer patients. 4) Anticoagulation therapy significantly ameliorated the patient's condition and mortality during ICU treatment.

D-dimer is a small protein fragment released into the blood when a blood clot is degraded by fibrinolysis (Adam et al., 2009). Thrombosis can be diagnosed by determining the D-dimer concentration: D-dimer is increased when the coagulation system is activated because of the associated thrombosis or disseminated intravascular coagulation. Of 1,643 patients with COVID-19 pneumonia, 691 showed high levels of D-dimer. The high level of D-dimer is a sign of deep venous thrombosis, pulmonary embolism, or disseminated intravascular coagulation (Ranasinghe and Bonser, 2010). A previous study reported that the morbidity of deep venous thrombosis was significantly decreased with the low level of D-dimer (<1.5 mg/L) (Kawaguchi et al., 2012). Zhou et al. found that high mortality in patients with COVID-19 was due to the high level of D-dimer (median: 5.2 mg/L). In our study, the elevated fibrinogen, PT percent activity, and decreased platelet usually accompanied the high level of D-dimer. Zhou et al. reported that the high level of D-dimer may help in the early clinical diagnosis of COVID-19 patients (Zhou et al., 2020). When the patients’ D-dimer was high, their median age was 65 years; about half of them showed fever/cough and respiratory symptoms. Increased creatinine and decreased albumin and hemoglobin were observed in the patients. This suggests that abnormal coagulation and kidney function may be associated with a high level of D-dimer. One reason for this phenomenon could be that the high levels of D-dimer induced abnormality in the coagulation system which could be associated with immune system damage. To our surprise, the elevated lymphocytes were observed in normal patients and elevated D-dimer patients after the increase in monocyte and interleukin-6, suggesting the activity of immune system in hospitalization. In this study, many patients were transferred from other hospitals after treatment for a period of time. Some of them have had the associated immune system. This maybe the reason for the elevated lymphocytes observed in patients in Leishenshan Hospital. Additionally, more patients displayed positive SARS-CoV-19 IgG, which also proved our hypothesis.

A study in most SARS patients has reported an association between thrombocytopenia and elevated D-dimer (Tan et al., 2005). Similarly, elevated D-dimer levels were also reported in patients with Ebola (Smither et al., 2019). Huang et al., (2020) reported that D-dimer values were nearly fivefold higher in those with severe disease caused by COVID-19 pneumonia. Recent literature data also showed that D-dimer values are frequently elevated in patients with COVID-19 (Lippi and Plebani, 2020). Tang et al. recently reported that the vast majority of COVID-19 patients who died during hospital stay fulfilled the criteria for diagnosing disseminated intravascular coagulation. Comorbidities that were present in COVID-19 patients increased the risk for mortality as reported in the current study (Zhou et al., 2020). Coronary heart disease and hypertension have been reported to be associated with poor outcomes in COVID-19 pneumonia and other respiratory viral infections (Corrales-Medina et al., 2013; Udell et al., 2013; Blackburn et al., 2018; Zhou et al., 2020). Diabetes has been reported to be a potential risk factor for the mortality of patients with COVID-19 infection (Yang et al., 2020). Liang et al. reported that patients with cancer were susceptible to COVID-19 pneumonia (Liang et al., 2020). In our study, of 691 patients with elevated D-dimer, 36.6% of them had cardiovascular disease, 12% had diabetes, less number of patients had cancer and other comorbidities. High levels of D-dimer have been associated with mortality in patients with infection in the emergency department (Rodelo et al., 2012). Both pro-coagulant factors and pro-inflammatory cytokines were essential for the induction of ischemia and thrombosis (Corrales-Medina et al., 2012). In this study, increased interleukin-6 level as well as higher monocyte and lymphocyte counts were found in elevated D-dimer patients.

Tis study has some limitations. First, patients admitted in Leishenshan Hospital were transferred from other designated hospitals; some patients did not receive standard supportive therapy and the medical history was not known. In addition, due to the time pressure of the epidemic outbreak, detail information such as objective data or detail information on treatments was not extracted from the from the electronic medical record. Second, some patients already received the relative antiviral therapy, which contributed to the poor clinical outcomes. Third, because of the retrospective study design, not all laboratory tests were performed in all patients, including IL-8, IL-10, blood glucose levels, and myocardial function. Fourth, we did not analyze all of cases in Leishenshan Hospital; 15 patients were not permitted to leave the hospital because of serious illness. Fifth, due to missing data, some patients were excluded from the analysis.

In summary, we found that a high level of D-dimer in patients with COVID-19 was associated with abnormal immunity, presence of comorbidities, disease severity, and increased mortality. D-dimer may be a potential prognostic marker for the early warning of severe cases. We believe that the findings are helpful for the treatment of the COVID-19 patients who showed elevated D-dimer.

## Data Availability Statement

The original contributions presented in the study are included in the article/Supplementary Material, further inquiries can be directed to the corresponding authors.

## Ethics Statement

The studies involving human participants were reviewed and approved by this study was approved by the Research Ethics Committee of Wuhan University Zhongnan Hospital (approval number 2020004). The patients/participants provided their oral consent to participate in this study. Oral informed consent was obtained from the individual(s) for the publication of any potentially identifiable images or data included in this article. Written consent was waived by the ethics committee since this infectious disease was rapidly evolving.

## Author Contributions

JL and ZL performed most of the study and participated in writing the original manuscript. MY and KL supervised the study and was involved in writing the results and discussion. GW involved in collecting original data and discussion. XX, LX and YC participated in data analysis. XW proposed the original idea and design of the study, supervised the study, and edited the manuscript. JC provided intellectual input and involved in discussion. QW participated in original idea and discussion.

## Funding

This study was supported by National Natural Science Foundation of China (NSFC) (81771280).

## Conflict of Interest

The authors declare that the research was conducted in the absence of any commercial or financial relationships that could be construed as a potential conflict of interest.
